# P-1481. Does Herpes Zoster ICD Coding with Complications Capture Clinical Severity of Shingles in Nursing Home Residents? A Case Review Study

**DOI:** 10.1093/ofid/ofaf695.1667

**Published:** 2026-01-11

**Authors:** Mriganka Singh, Yasin Abul, Thomas Bayer, H Edward Davidson, Lisa Han, Ivis Perez, Daniel Harris, Kaleen Hayes, Kevin McConeghy, Stefan Gravenstein

**Affiliations:** Alpert Medical School, Providence, Rhode Island; Brown University, Providence, Rhode Island; Providence VA Medical Center, Providence, Rhode Island; Insight Therapeutics LLC, Norfolk, Virginia; Insight Therapeutics, LLC, Norfolk, Virginia; Insight Theraputics, Norfolk, Virginia; University of Delaware, Newark, Delaware; Brown University, Providence, Rhode Island; COIN-LTSS, Providence Veterans Affairs Medical Center, Providence, Rhode Island; Brown University, Providence, Rhode Island

## Abstract

**Background:**

ICD codes identify when clinicians bill for herpes zoster (HZ) with or without complications. However, variation in billing and coding practices may limit administrative data’s utility classifying HZ and HZ severity in research applications. We identified and classified HZ severity in nursing home (NH) residents using clinical indicators extracted from electronic health records (EHR) of NH residents to assess the utility of HZ codes and EHR data sources for HZ research.

**Figure:**
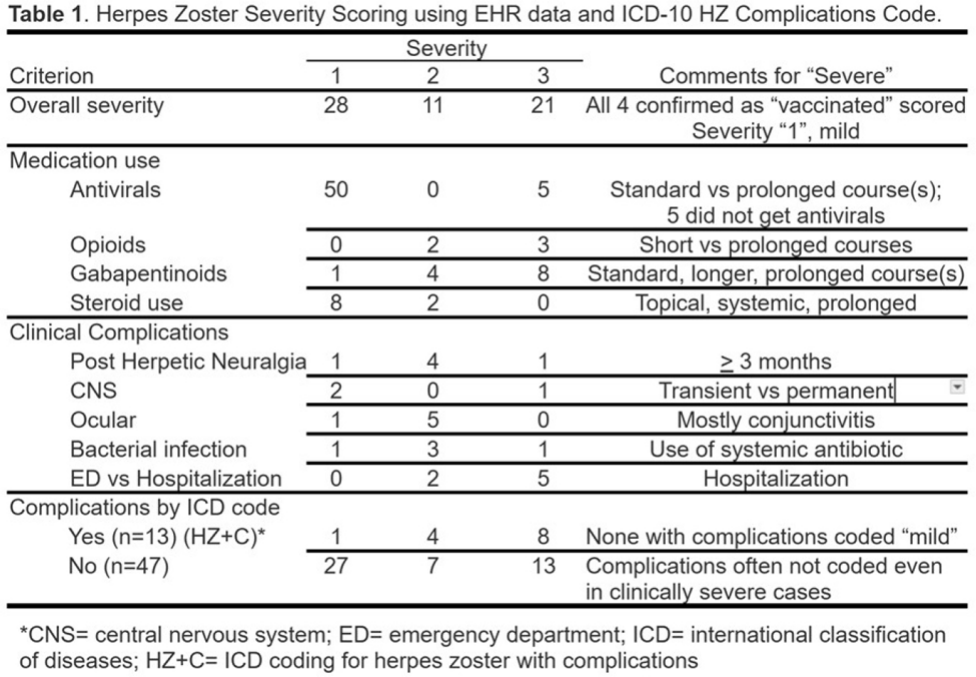

**Figure:**
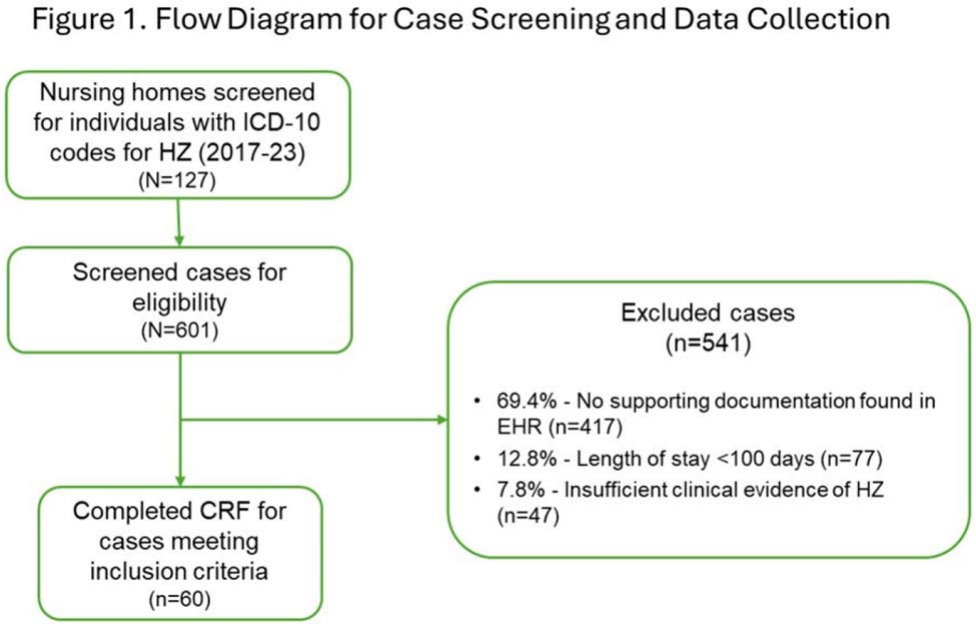

**Methods:**

We conducted case reviews on NH residents from a larger NH cohort study. Cases reviewed were identified by ICD-10 codes for HZ infection from the EHR of a 127-facility NH chain between 2017 and 2023. Cases were excluded (Figure 1) for no supporting documentation in the EHR, length of stay < 100 days, or lack of clinical evidence to support ICD code. The progress notes, medication administration records, and mandatory quarterly clinical assessments were systematically reviewed to complete case report forms (CRF) for data extraction. Three physicians reviewed and adjudicated tabulated CRF data.

**Results:**

From a population screened over 5 years, 601 cases were identified with possible HZ by ICD-10, 60 (cases) met inclusion criteria (Figure 1). Of cases, 74% were age 65 and over, 82% female, and 7% (n=4) vaccinated. Of these, 21 (35%) met a clinical severity rating (SR) of 3 (severe), and 11 (28%) were classified as moderate SR using a simple, internally developed rating scale. Of the 21 with SR of 3, 8 (38%) had ICD codes indicating HZ with complications (HZ+C), and 13 (62%) were coded without complications. Among those with SR of 2 (n=11, 18%), 4 (36%) had HZ+C (Table). Three hospitalized after HZ were not coded HZ+C. HZ rash was not often well-characterized in progress notes. Five individuals with HZ did not receive antiviral therapy.

**Conclusion:**

Severe outcomes were common, occurring in one third of HZ cases. ICD coding and clinical severity discordance in HZ cases highlights critical gaps in traditional surveillance methods, with 60% of cases leading to hospitalization not coded as severe. EHR-driven surveillance can improve detection of undercoded complications and enables a real-time burden assessment in long term care residents. Low case rate meeting inclusion criteria is a limitation.

**Disclosures:**

Mriganka Singh, MD, Glaxo Smith Kline: Grant/Research Support Yasin Abul, MD, CDC/ABT: Grant/Research Support|CLARIO: Advisor/Consultant|GSK: Grant/Research Support|Moderna: Grant/Research Support|Seqirus: Grant/Research Support H Edward Davidson, PharmD, GSK: Grant/Research Support|Moderna: Grant/Research Support|Sumitomo: Grant/Research Support Lisa Han, MPH, GSK: Grant/Research Support|Moderna: Grant/Research Support|Sumitomo: Grant/Research Support Ivis Perez, MPH, LPN, GSK: Grant/Research Support|Moderna: Grant/Research Support|Sumitomo: Grant/Research Support Daniel Harris, PhD, MPH, GSK: Grant/Research Support|Insight Therapeutics: Advisor/Consultant|Sanofi: Advisor/Consultant Kaleen Hayes, PharmD, PhD, Genentech: Grant/Research Support|GlaxoSmithKline: Grant/Research Support|Sanofi: Grant/Research Support Kevin McConeghy, Pharm.D., GlaxoSmithKline: Investigator-Initiated Study|Moderna: Grant/Research Support Stefan Gravenstein, MD, MPH, GSK: Advisor/Consultant|GSK: Grant/Research Support|GSK: Honoraria|Moderna: Grant/Research Support|Novavax: Advisor/Consultant|Novavax: Honoraria|Pfizer: Advisor/Consultant|Pfizer: Grant/Research Support|Pfizer: Honoraria|Sanofi: Advisor/Consultant|Sanofi: Grant/Research Support|Sanofi: Honoraria|Seqirus: Grant/Research Support

